# Using Continuous Glucose Monitoring as a Biological Feedback Strategy to Motivate Physical Activity in Cancer Survivors: A Mixed-Methods Pilot Study

**DOI:** 10.1177/10732748251359406

**Published:** 2025-07-28

**Authors:** Yue Liao, Grace E. Brannon, Chad D. Rethorst, Miranda Baum, Therese B. Bevers, Susan M. Schembre, Karen M. Basen-Engquist

**Affiliations:** 1 Department of Kinesiology, 2329 University of Texas at Arlington, Arlington, TX, USA; 2 Department of Communication, 2329 University of Texas at Arlington, Arlington, TX, USA; 3 199041 Texas A&M AgriLife Institute for Advancing Health Through Agriculture, Dallas, TX, USA; 4 Department of Health Disparities Research, 4002 University of Texas MD Anderson Cancer Center, Houston, TX, USA; 5 Department of Clinical Cancer Prevention, 4002 University of Texas MD Anderson Cancer Center, Houston, TX, USA; 6 6663 Georgetown Lombardi Comprehensive Cancer Center, Washington, DC, USA

**Keywords:** mHealth, digital health, biosensor, behavioral intervention, cancer survivorship

## Abstract

**Introduction:**

Identifying novel strategies to motivate regular physical activity in cancer survivors continues to be a critical mission, as the majority of cancer survivors are not sufficiently active to achieve the many health benefits of being regularly physically active. Providing biological feedback is one of the behavioral change techniques that shows promising effects in physical activity interventions. This study used a mixed-methods approach to test the acceptability and changes in physical activity motivation of a pilot intervention that provided personalized feedback via text messaging based on data from an activity tracker (Fitbit) and continuous glucose monitor (CGM) over a 4-week period.

**Methods:**

Twelve breast and colorectal cancer survivors completed this pilot intervention, which involved a one-on-one educational session followed by a 4-week intervention period with a Fitbit wristband and CGM. They received 2-3 weekly text messages based on their Fitbit and CGM data that aimed to increase their motivation to engage in physical activity. Participants completed surveys assessing motivational readiness before and after the intervention, and a post-intervention survey that assessed acceptability of the intervention. Exit interview was also conducted to collect their feedback and opinions toward the intervention.

**Results:**

Both quantitative and qualitative results suggest a high acceptability of the study devices (ie, Fitbit and CGM) as well as the intervention components (e.g., the glucose-based biological feedback). Participants reported a significant decrease in the preparation stage and an increase in the action and maintenance stages (ps < 0.05). Results from qualitative analysis further indicate participants’ positive changes in physical activity motivations.

**Conclusion:**

The use of CGM along with an activity tracker is a viable method to provide personally relevant and motivating biological feedback messages to motivate physical activity in cancer survivors. Future studies can incorporate this behavior change technique into their intervention and further evaluate its impact on behavior change and related health outcomes.

Clinical trial number: NCT05124405.

## Introduction

Regular physical activity is important for improving physical and mental health in cancer survivors and can reduce the risk of progression of several types of cancers.^1-3^ Despite broadly targeted public health efforts aimed at increasing physical activity and decreasing sedentary time, most cancer survivors remain insufficiently active in their daily life.^4^ Therefore, novel intervention strategies are needed to support sustainable physical activity behavioral change in cancer survivors. The goal of this study was to explore how we might leverage data from wearable biosensors to provide personalized biological feedback as a strategy to promote daily physical activity in cancer survivors.

Several behavior change theories and intervention frameworks emphasize the importance of increasing an individual’s motivation to promote the adoption and maintenance of healthy behaviors, such as physical activity.^5-7^ One behavioral change technique that is highly promising yet has been relatively underutilized in behavioral interventions is biological feedback. According to the behavior change technique taxonomy, biological feedback (or biofeedback) is defined as providing “feedback about body (e.g., physiological or biochemical state) using an external monitoring device as part of a behavior change strategy.”^8^ Although the concept of biological feedback is not new, how to best utilize and incorporate this personalized data into lifestyle change interventions remains an open question.^9^ With the rapid advancement in wearable sensing technology, there are huge potentials to use these continuously measured physiological and biological data to motivate behavioral changes in physical activity.^10^ For biological feedback to effectively motivate physical activity, the candidate biological metric needs to have long-term clinical implications while being sensitive to daily behavior changes in physical activity and have the technological capacity to be assessed continuously throughout the day. Therefore, we propose that glucose data, measured via continuous glucose monitors (CGMs), have great promise as a behavior change tool for cancer survivors for the following reasons. First, diabetes is not only one of the most common comorbidities in cancer survivors^11,12^ but also has been linked with increased cancer mortality, especially among those who are insufficiently physically active.^13^ Second, acute bouts of physical activity can improve insulin sensitivity and increase glucose uptake by skeletal muscles, and this immediate impact of physical activity on glucose has been well demonstrated in controlled lab environments among individuals without diabetes.^14-16^ Third, CGMs, which consist of a tiny sensor that goes under the skin, have seen rapid technological advancement in the past few years and are becoming more accessible in the consumer market.^10^ Data from CGMs can be linked to a mobile application to display real-time glucose data and historical trends for end-users, as well as can be wirelessly uploaded to cloud-based servers for third-party access (e.g., healthcare providers and interventionists).

The current pilot study aims to test the feasibility of using CGM as the basis for providing biological feedback to motivate physical activity in a sample of cancer survivors. In addition to the theoretical and scientific premise, this pilot intervention was informed by our qualitative work in the target population – cancer survivors who are at a higher risk of developing type 2 diabetes.^17^ Overall, findings from this formative work showed that cancer survivors found receiving personalized biological feedback based on their glucose data as highly acceptable, personally relevant, and motivating. As the first study to deliver CGM-based biological feedback to motivate physical activity in cancer survivors, the goals of this pilot intervention were to (1) determine the acceptability of a physical activity intervention that incorporated the use of CGMs in overweight and obese cancer survivors and (2) evaluate changes in exercise motivation, using both quantitative and qualitative approaches. A mixed methods approach was chosen to capture both the measurable acceptability and impact of the intervention on physical activity and motivation (quantitative data) and the nuanced, subjective experiences of participants (qualitative data) that influence acceptability and behavioral change. While objective metrics for acceptability and motivation allowed comparison with other studies that used similar measures, qualitative interviews provided a deeper understanding of how study participants perceived the personalized feedback, interpreted their data, and integrated these tools into their daily lives. This combination of quantitative and qualitative approaches enabled a more comprehensive evaluation of both real-world acceptability and preliminary efficacy of the intervention, which would not have been fully understood through either method alone.

## Materials and Methods

### Study Overview

The current pilot study was an expansion of the My Moves intervention, originally designed for adults with overweight/obesity but without diabetes.^18^ Revision of the My Moves intervention components was based on a focus group with the targeted patient population.^17^ This enhanced version of the intervention extended the original intervention period from 10 days to 28 days and added the personalized text messaging component. Upon study enrollment, participants completed a baseline survey and a one-on-one educational session that briefly overviewed the various benefits of physical activity, with information about the acute impact of physical activity on daily glucose patterns. Participants were also provided with a Fitbit Inspire wristband and 2 Freestyle Libre 14 Day CGM sensors to wear for the next 28 days (i.e., the intervention period). During the intervention period, participants can use the Fitbit and CGM smartphone apps to monitor their daily activity and glucose patterns. In addition, participants received 2 to 3 weekly text messages based on their Fitbit and CGM data that aimed to increase their motivation toward physical activity. At the end of the intervention period, participants completed the post-intervention survey and an exit interview. All study participants provided written informed consent. The study protocol was reviewed by the Clinical Research Committee and the Institutional Review Board at the University of Texas MD Anderson Cancer Center with a study protocol number 2018-0299 and an approval date of March 9, 2020. This study complies with the GRAMMS guidelines.^19^

### Participants

Adult patients with breast or colorectal cancer (stage I-III) were identified through MD Anderson’s tumor registry. Patients with a physical address in the surrounding areas of the Texas Medical Center in Houston, Texas, were contacted by email with study information. This email included a link to a web-based survey for individuals to indicate reason(s) not interested in this study. Study information was also shared on social media as well as through MD Anderson’s Center for Energy Balance’s newsletter and webpage. Interested individuals were asked to contact the study team to obtain a more detailed description of the study, which included a brief introduction to the CGM. Individuals who remained interested were sent an online screening questionnaire to determine their initial eligibility.

Eligible individuals were men and women aged 18 to 65 years who self-reported as having been diagnosed with stage I-III breast or colorectal cancer and completed adjuvant therapy; had a BMI ≥25 kg/m^2^; engaged in less than 150 minutes of moderate-intensity physical activity per week in the previous month; could speak, read, and write English; and had a smartphone phone with daily internet access that was CGM compatible. Individuals were excluded if they reported being diagnosed with diabetes; reported using any medication known to affect glucose levels (e.g., corticosteroids, antidepressants, metformin); reported health issues that might limit unsupervised physical activity; worked overnight shifts; were pregnant or lactating; were following a low-carb/ketogenic diet; or were unable or unwilling to use a CGM.

Individuals who passed the initial eligibility screening were scheduled for an in-person visit at MD Anderson after an overnight fast. Eligibility criteria based on BMI and diabetes status were confirmed with measured height and weight and a fasting blood glucose level <125 mg/dL (assessed with a commercially available glucometer). All study participants signed the consent form after a study staff provided an overview of the study with a PowerPoint presentation. Our recruitment goal was 10-15 participants, similar to other mHealth/digital health pilot intervention studies that used a mixed-methods approach that focused on feasibility and usability.^20-22^

### Baseline Visit

Upon study enrollment, participants first completed a set of web-based surveys that assessed demographic information and exercise motivations. They were then given a Fitbit Inspire (Fitbit Inc, San Francisco, California, USA) wristband to wear, which continuously tracks steps and activity levels with heart rate monitoring. Study staff assisted participants with downloading the Fitbit application to their mobile phones and setting up the device. Participants were instructed to wear the Fitbit at all times, including while showering and sleeping, and to regularly sync it with the phone application. After the introduction to Fitbit, study staff went over a physical activity education session with the participant, which is detailed elsewhere.^18^ Briefly, the physical activity education session highlighted the immediate impact physical activity can have on glucose levels with handouts and an interactive web-based simulator. Participants were encouraged to observe this dynamic relationship between physical activity and glucose during their self-monitoring period using CGM and Fitbit (e.g., when seeing high glucose readings, try to engage in some physical activity and see how it might have impacted the glucose levels).

Following the education session, participants were given a FreeStyle Libre 14-day CGM system (Abbott Diabetes Care Inc, Alameda, California, USA), which consists of a sensor (measures at 5 mm height and 35 mm diameter, and weighs 5 grams) and a smartphone app (FreeStyle LibreLink). Study staff helped participants insert the sensor into the back of their upper arm and download the app to their phone. All sensors were calibrated at the time of insertion, using a commercial glucometer with a finger prick. Upon activation, the sensor records interstitial glucose data every 15 minutes continuously for 14 days without the need for finger-stick calibration. Participants were provided an additional sensor to be replaced at the end of the first 14 days, for a total of 28 days CGM monitoring. To ensure the sensor stayed in place, participants were advised to avoid activities that would submerge the sensor under water for a long period of time, including baths and swimming.

### Intervention Period

#### Self-Monitoring

The 28-day (4-week) intervention period started the day after the one-on-one physical activity education session. During this period, participants could check their cumulative steps and activity levels at any time through the Fitbit application. Participants were reminded about checking their heart rate readings from the Fitbit wristband while exercising to make sure that the activity reached moderate intensity. Participants were also instructed to check their glucose information in using the LibreLink app by scanning the sensor with their smartphone. Upon scanning, the LibreLink app displays the current glucose reading and a graph showing 8 hours of glucose history, with an arrow indicating the directional trend. For self-monitoring purposes, participants were asked to obtain a glucose reading at least 4 times a day: each morning when they woke up, each night before they went to sleep, and at least 2 other times throughout the day (e.g., after lunch and after dinner). Participants received 4 reminders each day through text messages for the scanning. Participants were encouraged to observe how their daily glucose patterns were influenced by their behaviors (e.g., eating and exercising). We explained to participants that, as a person without diabetes, it is normal to see glucose levels go up and down throughout the day, depending on food intake and physical activity levels. Participants were suggested to consult their primary physician if they had any concerns regarding their glucose readings.

#### Personalized Text-Messaging

During the 4-week intervention period, participants received feedback that integrated data from their Fitbit device and CGM by 2-4 text messages each week. The development of these intervention messages was based on feedback from a focus group study in breast and colorectal cancer survivors^17^ with the goal of providing personalized biological feedback to motivate physical activity. A total of 13 personalized feedback messages were scheduled (9 messages were based on Fitbit data, and 4 messages were based on CGM data) to be delivered over the 4-week period. Messages were based on the social cognitive theory^23^ and the health belief model,^24^ incorporating constructs such as goal setting, self-monitoring, outcome expectation, somatic sensations, social support, and cue to action. All text messages were managed and delivered through Mosio (Mosio Mobile Messaging, Seattle, Washington, USA), a text messaging software platform for clinical research.

Fitbit-based feedback messages were sent out automatically based on participants’ Fitbit data. Participants’ real-time Fitbit data and device status (ie, battery and syncing) information were captured by Fitabase (Small Steps Labs LLC, San Diego, California, USA), a web-based platform that processes Fitbit data and generates reports. Mosio obtained Fitbit data from Fitabase’s application programming interface (API). Several physical activity metrics were calculated based on the Fitbit data, including total weekly active minutes, total daily active minutes, and total daily step counts. An algorithm was set up so that participants received different messages based on their performance towards a goal (ie, if meeting the weekly goal of accumulating 150 active minutes) and if they had made progress towards the goal (ie, if there was an increase or decrease in the weekly active minutes compared to the previous week).

CGM-based feedback messages were sent out manually by a study staff upon reviewing participants’ CGM data via the LibreView web portal, a cloud-based system provided by Abbott for healthcare professionals to obtain reports from the FreeStyle glucose monitoring devices. Participants’ real-time CGM data was synced to the LibreView portal when they used their smartphone to scan the sensor. Study staff obtained weekly average glucose level and weekly accumulated time spent above 180 mg/dL upon checking each participant’s glucose report once a week and using that information to send out the CGM-based feedback messages. We included sample personalized feedback messages in the Supplemental Table.

### Post-Intervention Visit

Participants completed the post-intervention survey and an exit interview after the 4-week intervention period. The exit interview followed a semi-structured interview guide that aimed to capture participants’ acceptability of wearing the wearable sensors and receiving Fitbit/CGM-based feedback. All interviews were audio recorded, lasting approximately 60-90 minutes, and transcripts were transcribed and verified by members of the research team. Participants received up to $40 in gift cards and kept their Fitbit device for completing this study.

### Measures

#### Acceptability

A 10-item survey was used to assess the acceptability of wearing the Fitbit and the CGM during the intervention period, as in previous studies.^18,25^ The survey items focused on addressing the barriers and facilitators to the use of mHealth tools, such as convenience, value, and relevance. The response options were rated using a 5-point Likert scale, ranging from “strongly disagree” to “strongly agree.”

#### Exercise Motivation

The Behavioral Regulation in Exercise Questionnaire-2 (BREQ-2) and the Exercise Stages of Change – Continuous Measure (URICA-E2) were used to assess exercise motivation. The BREQ-2 captured the stages of the self-determination continuum, including amotivation, external regulation, introjected regulation, identified regulation, and intrinsic regulation, reflecting constructs in the self-determination theory.^26^ The responses were rated on a 5-point Likert scale, ranging from “not true” to “very true.” The URICA-E2 captured readiness to change, as described in the transtheoretical model, in 6 stages: pre-contemplation (non-believer), pre-contemplation (believer), contemplation, preparation, action, and maintenance, with the assumption that physical activity behavior is typically a dynamic process that moves between different stages.^27^ The responses were rated using a 5-point Likert scale, ranging from “strongly disagree” to “strongly agree.”

### Statistical Analysis

Descriptive statistics were generated for all variables, including the means and standard deviations for continuous variables and percentages for categorical variables. A summary score of acceptability was created for Fitbit and CGM by calculating the mean of the 10 survey items, as in previous studies.^18,25^ Two-tailed paired-sample *t*-tests were used to examine changes in exercise motivations and biomarkers before and after the intervention. A *P*-value of .05 or less was considered statistically significant. All statistical analyses were conducted using SPSS software (IBM Corp, Armonk, NY, USA) version 24.0.

### Qualitative Analysis

Integration of quantitative data and qualitative data was integral to this project. Specifically, members of the research team relied on Braun and Clarke’s six-step process to manage data collection, processing, and analysis, which was informed by the quantitative analyses.^28^ To familiarize with the data, research team members read the transcripts. Following data familiarization, members of the team created initial coding categories and related subcategories. Team members involved in the coding met weekly during the category creation period to resolve differences related to coding or interpretation by discussion. After, themes were created based on the developed categories and subcategories, with relevant exemplars selected for each theme. Research team members then reread the transcripts to ensure that the data was fully analyzed. The themes are discussed with relevant participant quotes.

## Results

### Participant Characteristics

**Figure 1.**
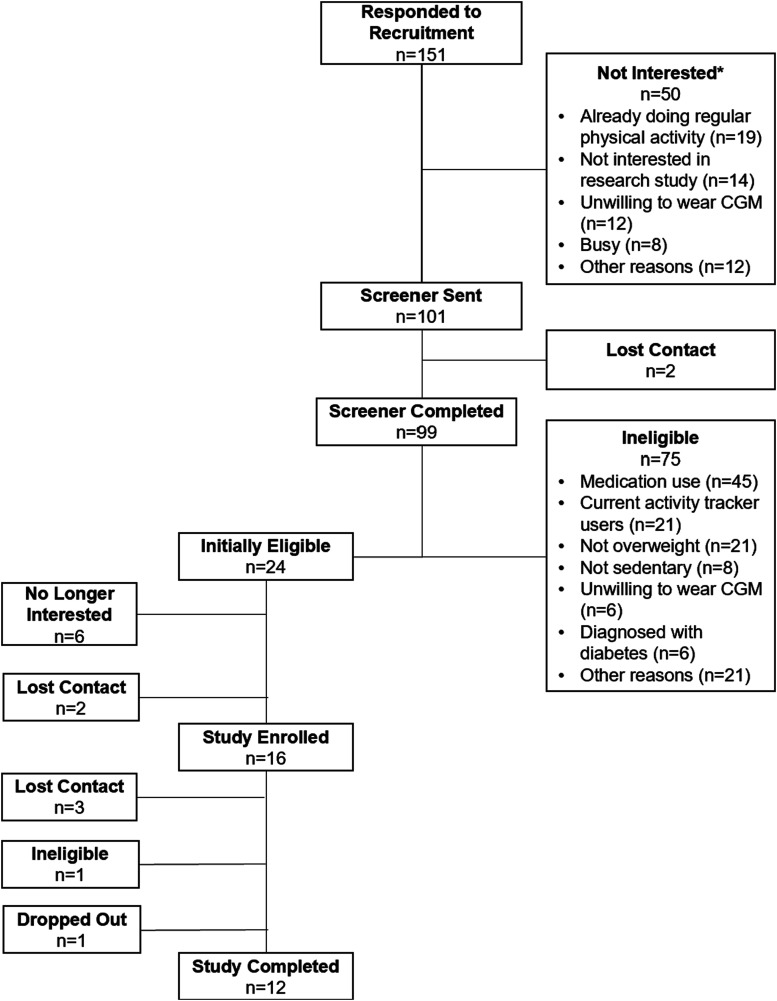
Study Participants Flowchart.

Figure 1 summarizes the flow of participants from recruitment to study completion. A total of 151 individuals responded to our recruitment efforts. Fifty responded by indicating they are not interested in the study. Among these, 19 disclosed that they were already doing regular physical activity, 14 mentioned that they were not interested in doing any research study, 12 indicated they did not want to wear the CGM sensor, 8 mentioned they were too busy, and 12 mentioned some other reasons (e.g., self-determined that they were not eligible for the study). Eligibility screener was sent out to the 101 individuals who were interested in this study, and 99 individuals completed the screener. Of these, 75 were ineligible for the study, mostly due to medication use (e.g., corticosteroids, metformin, n = 45). A total of 24 individuals were eligible on the basis of their screener answers. Of these, 6 were no longer interested in participating and 2 were lost contact. A total of 16 individuals signed the consent form and enrolled in the study. Of these, 3 lost contact and did not start the intervention, one was no longer eligible after fasting glucose test, and one dropped out of the study after starting the intervention. A total of 12 participants completed the study and were included in the analysis.

Table 1 shows the demographic characteristics of the 12 participants. Participants’ average age was 52 years (SD = 11.9, range 35-75). Majority (11/12) of the participants were female, 42% (5/12) were from a racial minority group. Most participants (8/12) had a college degree or above. Participants’ average fasting glucose at baseline was 85.9 mg/dL (SD = 4.5, range = 78-95).

**Table 1.** Participant Characteristics (n = 12)

Characteristic	No. of participants (%)^a^
Mean age (SD), years	51.6 (11.9)
Race/ethnicity	
African American	3 (25.0)
Asian	2 (16.7)
Hispanic white	2 (16.7)
Non-Hispanic white	5 (41.6)
Education level	
Some college/technical training	4 (33.3)
Bachelors degree	6 (50.0)
Masters degree	2 (16.7)
Marital status	
Single	4 (33.3)
Married/living with significant other	5 (41.7)
Divorced/separated/widowed	3 (25.0)
Employment status	
Employed (full-time)	8 (66.7)
Not employed	2 (16.7)
Retired	2 (16.7)

SD, standard deviation.

^**a**^All data are no. of patients (%) unless otherwise indicated.

### Feasibility and Acceptability

#### Quantitative Measures

Participants did not report any major problems with wearing the Fitbit or the CGM sensors during the intervention period. Participants on average wore the Fitbit device on 97% of the days out of the intervention period (ranged from 86% to 100%). A valid Fitbit wear day was defined as wearing the Fitbit for at least 10 hours during the waking hours. On average, the CGM sensor stayed active (ie, was able to collect and store glucose data every 15 minutes) 84% of the time during the 28-day period (SD = .14, range = 54%-100%). Supplemental Figure 1 shows the weekly average frequency of hyperglycemia events (i.e., incidents when glucose readings were greater than 140 mg/dL). Participants, on average, scanned the sensor with their smartphone 7 times a day (SD = 3.7, range = 3-17). Supplemental Figure 2 shows participants’ daily step counts and active minutes captured by Fitbit, along with the type of message being sent that day (i.e., personalized feedback message based on Fitbit data vs personalized biological feedback message based on CGM data).

For the Fitbit device, all participants agreed with statements regarding usability, convenience, value, relevance, tech support, recommendability, and likability. Eighty-three percent (10/12) agreed with the statement regarding motivating, ninety-two percent (11/12) agreed with the statement regarding confidence, and 17% (2/12) expressed concerns about privacy. For the CGM, all participants agreed with statements regarding usability, relevance, tech support, and confidence; 92% (11/12) with convenience, value, and recommendability; 83% (10/12) with motivating and likability; and 17% (2/12) expressed concern about privacy. Overall, the Fitbit had an acceptability score of 4.43 (SD = 0.27), and the CGM had an acceptability score of 4.36 (SD = 0.28). Table 2 shows the average score for each acceptability item.

**Table 2.** Participants’ Experiences With the Wearable Sensors During a 28-Day Intervention Period (n = 12)

Acceptability survey item	Mean likert rating^a^ (SD)	
	Fitbit	CGM
Usability: This tool is easy to use and user friendly	4.42 (0.51)	4.58 (0.67)
Convenience: This tool is convenient for me to use in my everyday life	4.50 (0.52)	4.25 (0.87)
Value: This tool is useful and beneficial	4.58 (0.51)	4.58 (0.67)
Relevance: This tool provides information that is of interest to me	4.58 (0.51)	4.58 (0.67)
Motivating: I am motivated to use this tool to track my daily behaviors	4.25 (1.22)	4.17 (1.19)
Tech support: There is adequate availability and quality of professional assistance throughout use of this tool	4.58 (0.51)	4.64 (1.42)
Confidence: I Feel confident that I use this tool correctly	4.33 (0.89)	4.50 (0.67)
Privacy: I am concerned about my privacy when using this tool	2.25 (1.54)	2.25 (1.54)
Recommendability: I would recommend this tool to my friends and family	4.58 (0.51)	4.33 (0.78)
Likeability: I like using this tool	4.67 (0.49)	4.16 (1.03)

SD, standard deviation.

CGM, continuous glucose monitor.

^a^The Likert scale used in the ratings was 1 = strongly disagree, 2 = disagree, 3 = neither agree nor disagree, 4 = agree, 5 = strongly agree.

#### Qualitative Interviews

Five primary themes emerged from the data: study device usability and convenience, wearable data value and relevance, motivation to use the study device(s), intervention messaging acceptability, and future intervention suggestions. Overall, the study devices (CGM and Fitbit), which facilitated the idea of understanding the acute impact of physical activity and diet on glucose levels, were well received by participants. This suggests that people without diabetes can understand and find value in glucose-based biological information; some even planned behavior changes based on this information.

##### Device Usability and Convenience

When asked if they found the heart rate information from the Fitbit to be useful when they exercise, all but one of the participants said yes. When thinking about wearing CGM sensor, the only negatives were some occasional soreness, some tackiness from residual adhesive when removing the sensor, and one participant noted that wearing the CGM “didn’t work” for some of her preferred outfits.

##### Wearable Data Value and Relevance

Participants identified several relevant Fitbit features when asked how they felt about the information from the app. These included hours of sleep, water, steps, active minutes, days exercised, step count, and heart rate values. Participants reported having a better understanding of glucose averages and trends through the use of CGM. When asked about the relevance of CGM data, participants described that the data from the CGM allowed them to make connections with their current physical state (e.g., a headache) or that specific meals were more likely to cause spikes in glucose levels. All but one of the participants indicated that the frequency of receiving glucose-related intervention messages was valuable. Participant 002 said that it was through these text messages that they learned about the ideal glucose values.

##### Motivation

Because the Fitbit devices report heart rate targets, nine of the participants said that they used that information to try to hit their targets when exercising. Seven of the twelve reported that knowing their glucose numbers motivated them to change their diet, while eleven indicated that they used that number to change their physical activity behaviors (as intended by the intervention messages). When asked which device (Fitbit or CGM) motivates them more to exercise, six said both the same, five said Fitbit, and one said CGM.

##### Intervention Messaging Acceptability

Participants were positive or neutral about the intervention messaging, primarily reporting that they were relevant and provided important information. Specifically, one participant said, “The messages were very encouraging; [it] felt like someone was advising me. [They] provided small ways to improve.” Others said they provided timely reminders to exercise. Three indicated that while some of the information received in the text messages could be found elsewhere (e.g., the Fitbit app), it was helpful to have it at one’s fingertips rather than taking the time to search through the app; this indicates that participants like having information delivered in multiple ways, increasing perceptions of accessibility.

##### Future Intervention Suggestions

Participants provided suggestions for future interventions, including their perceptions of the CGM-sensor element of the intervention being 4 weeks long. Nine of the twelve felt the intervention period was “too short” while the remaining 3 perceived that the intervention period was “about right.” Participants explained their answers, saying that habits take time to implement, especially since, “Feels like 2 months or more would be better because the first month you’re figuring everything out” (014) and “Once I understood how everything was working with the study, the study was already over” (008). Another (016) appreciated the “accountability” and “security” that the sensors provided. One participant who felt the intervention period was about right stated, “It gives enough time to see your patterns (exercise, eating, and glucose levels)” (010). No participants indicated that the intervention period was too long. Participants provided ranges of how many days would be better, with responses from 6 weeks to several months reported.

Participants also indicated that other kinds of information or feedback they wished they could have received related to their CGM data. These responses included more details in trend lines (002), information to know what “foods or exercise” would raise glucose levels if they were low (003), and an indication if the glucose level was too high (009). Seven participants did not report wanting anything additional related to the CGM data.

### Exercise Motivation

#### Quantitative Measures

Table 3 shows the changes in exercise motivation before and after the intervention period. Overall, there was no significant change in self-determination motivation. For stages of change, at visit 1, participants scored higher for the contemplation and preparation stages compared to the other stages, suggesting that most of them were considering a change in their physical activity behavior. After the intervention period, the score decreased significantly for the preparation stage (*P* = .029) and increased significantly for the action and maintenance stage (*P* < .05).

**Table 3.** Changes in Exercise Motivations Before (Visit 1) and After (Visit 2) the Intervention Period

Motivational scale	Mean likert rating (SD)		Change (mean, SD)	*p*-value^a^
	Visit 1	Visit 2		
Self-determination motivation^b^				
Amotivation	0.02 (0.07)	0.00 (0.00)	−0.02 (0.07)	.339
External regulation	0.40 (0.74)	0.50 (0.83)	0.10 (0.52)	.499
Introjected regulation	2.53 (0.92)	2.39 (0.92)	−0.14 (1.11)	.672
Identified regulation	3.56 (0.56)	3.53 (0.56)	−0.03 (0.33)	.777
Intrinsic regulation	2.73 (0.87)	2.54 (0.73)	−0.19 (0.95)	.508
Stages of change^c^				
Precontemplation (non-believers)	1.25 (0.37)	1.10 (0.20)	−0.15 (0.27)	.089
Precontemplation (believers)	1.83 (0.80)	2.06 (0.89)	0.23 (0.86)	.377
Contemplation	4.00 (0.89)	3.75 (0.94)	−0.25 (0.70)	.241
Preparation	3.15 (1.12)	2.44 (0.69)	−0.71 (0.98)	.029
Action	2.60 (0.92)	3.75 (0.99)	1.15 (1.35)	.013
Maintenance	2.38 (1.08)	3.15 (0.76)	0.77 (0.78)	.006

SD, standard deviation.

^a^Significance test using the two-tailed paired-sample *t* test.

^b^The Behavioral Regulation in Exercise Questionnaire-2 (BREQ-2). The Likert scale used in the ratings ranged from not true (0) to very true (4).

^c^The Exercise Stages of Change – Continuous Measure (URICA-E2). The Likert scale used in the ratings ranged from strongly disagree (1) to strongly agree (5).

#### Qualitative Interviews

While there were no significant quantitative changes in self-determination motivation, participants did qualitatively reflect on their motivation to monitor their physical activity patterns and glucose information and were introspective about how the 2 may relate, as evidenced by the corpus of interview data. Specifically, participants indicated that the data from both Fitbit and CGM devices increased their motivation to engage in physical activity by increasing their daily step count. They also indicated this knowledge helped with goal setting and achieving their goals. For example, participant 006 talked about the knowledge gained from using the Fitbit during the study as related to physical activity patterns, “I’ve learned that exercise is beneficial and the more you exercise the more you can regulate your levels… It’s beneficial knowing how many steps you’re taking a day, it’s making you aware if you’re being active or being active enough.”

Secondly, they said that the CGM data helped encourage thinking about food and diet decisions, which motivated them to reevaluate certain diet decisions. For example, 010 noted that, “… sometimes at work, I would get busy and we take a long time to eat lunch, I noticed I would start going low and I would kinda get a headache, and then my numbers were low, I knew that I need to eat sooner...” Likewise, participant 014 described how incorporating protein and cutting back on snacking was now a part of their lifestyle, with an overall increase in awareness. The participant added, “I have a long way to go, but I was eating like a light salad a couple of days in a row, but not a lot of dressing, a lot of greens on it, so... I'm more aware and I'm making the changes...” Participant 003 said, “It [seeing your CGM data] affects your [food] choices because when you see now, we want to be something to make you identify, want to refrain from eating certain things.” 008 said, “Yes, I'm eating more salads. Drinking more water. Let's see, I'm trying not to eat so late in the evening and finding alternatives if I go to a fast-food restaurant…” like consuming only half the meal.

Thirdly, several individuals commented that they saw positive biological changes that helped further motivate them to work towards their goals. For example, several participants gave the example of their resting heart rate being lower than before engaging in the study. Participants also reported learning about their health habits throughout the study and further indicated that they perceived takeaways from their participation that they could continue to carry over into their daily lives. These lessons centered on mindfulness and encompassed topics from physical activity patterns, glucose patterns, and diet, plus combinations of these topics together. Participant 010 felt that having the CGM data provided explanations for what was occurring biophysically, saying, “It was just like a visual representation to kind of help you [understand]... It was easier to understand.”

## Discussion

To our knowledge, the present study was among the first that utilized CGM to provide personalized biological feedback messages in cancer survivors to motivate physical activity. The main focus of this study was to collect feasibility and usability data in real-life settings as we incorporated novel technology-based intervention components into a text message-based physical activity intervention over 4 weeks. Overall, there were no major technical challenges or technical failures experienced by the study team or the study participants. Participants were able to re-apply the CGM sensors on their own, and all personalized feedback messages were able to be generated and delivered.

Both our qualitative and quantitative data supported the high acceptability in cancer survivors for the wearable sensors and the glucose-based biological feedback messages. The qualitative results regarding the acceptability of CGM sensors are consistent with previous studies in overweight and obese adults without diabetes,^18,25^ providing further support for the feasibility of using CGM devices in physical activity and behavioral intervention in populations without diabetes. In particular, the mean age of the current study participants was older (52 years old) than the previous studies (38 years and 42 years old), with the oldest being 75 years old. Our results demonstrate the wearable sensor technology could be acceptable in a wide age range. Qualitatively, participants did not report a lack of perceived confidence, a lack of value, or that the wearable sensors or the data were irrelevant to them. Rather, they reported that the data from the Fitbit and CGM sensors motivated them to take more steps, to meet goals (both step-related or glucose-level related) and think about other health-related behaviors such as nutritional decisions, and indicated that they’d seen positive changes (e.g., lower resting heart rate) that further encouraged them to continue working towards meeting their goals. With the continuous advancement in CGM technology (e.g., continuously passive glucose data collection without the need for active scanning, potentials for non-invasive sensors that can be embedded in a smartwatch), we anticipate the acceptability of CGM as a biological sensor will be even further enhanced in the broader population.

Similar to the original My Moves intervention in overweight and obese adults, we observed significant changes in exercise motivation in the stages of change scale in the current study. Notably, while both studies observed a significant increase in the “action” stage, only the current study also observed significant increases in the “maintenance” stage. This could be due to the fact that the current intervention was 4-week long, while the original My Moves was only a 10-day intervention. Since behavioral change maintenance has been a challenge across all behavioral interventions, our preliminary findings suggest that personalized biological feedback messages could be a promising strategy to promote physical activity maintenance. Coupled with the qualitative findings that two-thirds of the study participants felt the intervention period was “too short,” future CGM intervention could extend the study duration to further examine physical activity behavioral change maintenance.

The current study also did not see a significant change in the quantitative self-determination motivation scale as the original My Moves intervention. Nevertheless, results from our qualitative analysis touched on several key constructs of the self-determination theory. For example, *introjection* (ego involvement, focus on approval from self and others) was found through participants comparing themselves with their friends’ achieving their own goals (005), or focused on how they looked based on their weight loss during their involvement in the study (009). Several participants reported that there was a family health history of type 1 or type 2 diabetes, which caused them to have some “paranoia” (002) and motivated them to set proper nutritional and physical activity goals (006). *Identification* (e.g., personal importance, conscious valuing of activity, self-endorsement of goals) was also present. Having their glucose information provided, which they didn’t have easy access to previously, led to rumination on their health behaviors, which later led to specific goal setting and action beyond rumination. Participants exemplified that they have identified nutrition as important and self-endorsed their goals. This category of findings was where some participants were starting to link activity and glucose levels, or diet and glucose levels, together, but had not yet synthesized the relationship between these variables. *Integration* (e.g., congruence, synthesis, and consistency of identifications) was less common but still reported by participants. This was primarily exemplified by participants who made the connections between physical activity, diet, and glucose management. For example, 009 linked activity, diet, and glucose management together as a result of participating in the study, describing that, “If I exercise after eating, then I can help the curb of the [glucose level] increase, so I try to eat… I exercise in the morning anyway, but I try to do a few minutes of walking after eating.” Lastly, as participants moved towards *internalization* (e.g., interest, enjoyment, inherent satisfaction), they seemed to ruminate on their health behaviors (e.g., physical activity patterns and blood glucose levels) more clearly, which motivated them to subsequently change those behaviors. Of note is that 4 participants clearly stated that they enjoyed participating in the research study, as they learned a lot, thereby motivating them to continue engaging in healthy behaviors like eating a nutritious diet and engaging in physical activity. The participants did not report that they exercised purely for the inherent satisfaction of engaging in the activity itself but did enjoy the byproducts (e.g., lower resting heart rate) that they noticed as a result of their activity.

In sum, this pilot study provided quantitative and qualitative data to support the delivery of glucose-based biological feedback messages to cancer survivors to motivate their physical activity behavioral changes. Our in-depth qualitative results on CGM feasibility and self-reflection on behavioral change motivation offer additional insights into the design of future interventions that incorporate glucose-based biological feedback. For example, participants were able to link the connections between their daily glucose patterns and their behaviors through self-monitoring and appreciated the actionable behavioral change advice they were getting through the intervention text messages. The current study only sent CGM-based feedback once a week, future studies can increase the frequency of providing this personalized biological feedback so that it can be more timely and the intervention content can be more adaptive based on each individual’s own trajectory of change. Although the current study was not powered to detect changes in physical activity, data from the Fitbit device showed variations in changes in daily steps across the intervention period (see Supplemental Figure 2). This preliminary data provides some evidence to support the need for a more dynamic intervention content delivery strategy so that the feedback messages can be more tailored to each individual’s behavioral pattern. One important note is that, in the current study, participants, on average, scanned the CGM sensor using their phones to retrieve their glucose information 7 times a day. This personal interaction with the CGM sensor is no longer a requirement for newer models (e.g., FreeStyle Libre 3), where glucose data is passively transferred to a smartphone, and no scanning is needed. While these newer CGM models allow a more seamless data transfer between sensor and mobile device, future interventions need to consider how this passive data collection may impact self-monitoring. We believe this is why we cannot simply just provide a wearable sensor tool (such as CGM) to individuals and expect them to make behavior changes purely through self-monitoring. Actionable and timely feedback messages based on these wearable sensor data utilizing behavioral change techniques will be the key to effective interventions.^29^ Lastly, the current intervention was focused on motivating physical activity. Our qualitative results indicated that participants were also making observations regarding eating behaviors. Future interventions can consider the feasibility of providing more comprehensive lifestyle modification suggestions that target multiple health behaviors.

While this study used a mixed-methods approach to examine the feasibility and acceptability of a novel intervention strategy that utilized glucose-based biological feedback in cancer survivors, it has several limitations. Similar to most behavioral/lifestyle intervention studies that rely on volunteers, there could potentially be recruitment bias where cancer survivors who signed up to participate in this study were already motivated to make a change. This may partially explain why we did not observe a significant change in the quantitative self-determination motivation scale. Nevertheless, even for highly motivated individuals, making a sustainable lifestyle change may need additional support beyond the initial “thinking about making a change” stage. And our quantitative results from the stages of change scale further demonstrated that our intervention approach was able to make a positive impact on cancer survivors’ preparation, action, and maintenance stages. One limitation of the quantitative data collected from the Fitbit and CGM is that it may not fully capture the contextual or emotional factors influencing participants’ motivation, which the qualitative interviews aimed to explore; however, participants’ self-reports may have been influenced by social desirability bias, potentially shaped by their awareness of being monitored via wearable devices. Additionally, this study had a small sample of English-speaking breast and colorectal cancer survivors; therefore, our results might be limited due to the nature of this study sample. Future studies can further investigate the effect of glucose-based biological feedback on behavior change in a more diverse, adequately powered sample.

Overall, given the high acceptability of glucose-based biological feedback in the current study and the promising preliminary results in increasing behavioral motivation, we encourage future studies to consider how they may be able to integrate personalized biological feedback into their intervention components to provide more adaptive and effective behavioral change interventions.

## Supplemental Material

Supplemental Material - Using Continuous Glucose Monitoring as a Biological Feedback Strategy to Motivate Physical Activity in Cancer Survivors: A Mixed-Methods Pilot Study Supplemental Material for Using Continuous Glucose Monitoring as a Biological Feedback Strategy to Motivate Physical Activity in Cancer Survivors: A Mixed-Methods Pilot Study by Yue Liao, Grace E. Brannon, Chad Rethorst, Miranda Baum, Therese B. Bevers, Susan M. Schembre and Karen M. Basen-Engquist in Cancer Control

Supplemental Material - Using Continuous Glucose Monitoring as a Biological Feedback Strategy to Motivate Physical Activity in Cancer Survivors: A Mixed-Methods Pilot Study Supplemental Material for Using Continuous Glucose Monitoring as a Biological Feedback Strategy to Motivate Physical Activity in Cancer Survivors: A Mixed-Methods Pilot Study by Yue Liao, Grace E. Brannon, Chad Rethorst, Miranda Baum, Therese B. Bevers, Susan M. Schembre and Karen M. Basen-Engquist in Cancer Control

Supplemental Material - Using Continuous Glucose Monitoring as a Biological Feedback Strategy to Motivate Physical Activity in Cancer Survivors: A Mixed-Methods Pilot Study Supplemental Material for Using Continuous Glucose Monitoring as a Biological Feedback Strategy to Motivate Physical Activity in Cancer Survivors: A Mixed-Methods Pilot Study by Yue Liao, Grace E. Brannon, Chad Rethorst, Miranda Baum, Therese B. Bevers, Susan M. Schembre and Karen M. Basen-Engquist in Cancer Control

## Data Availability

De-identified data from this study can be available upon request from other researchers. *
