# Supplementary material for: Using Continuous Glucose Monitoring as a Biological Feedback Strategy to Motivate Physical Activity in Cancer Survivors: A Mixed-Methods Pilot Study
Source: Cancer Control. 2025 Jul 28;32:10732748251359406. doi: 10.1177/10732748251359406 (PMC12304624; doi:10.1177/10732748251359406)
Supplement: Supplemental Material - Using Continuous Glucose Monitoring as a Biological Feedback Strategy to Motivate Physical Activity in Cancer Survivors: A Mixed-Methods Pilot Study [file sj-pdf-1-ccx-10.1177_10732748251359406.pdf]

Study Evaluation

Please complete the survey below.

Thank you!

Please rate each of the following statement regarding the information you received during your first visit:

The _____ helped me better understand the health benefits of physical activity

	Not applicable	Strongly disagree	Disagree	Neither agree nor disagree	Agree	Strongly agree
Handout	◯	◯	◯	◯	◯	◯
Glucose simulator	◯	◯	◯	◯	◯	◯
Personal heart rate target calculation	◯	◯	◯	◯	◯	◯
Exercise plan	◯	◯	◯	◯	◯	◯

The _____ increased my motivation to be more active

	Not applicable	Strongly disagree	Disagree	Neither agree nor disagree	Agree	Strongly agree
Handout	◯	◯	◯	◯	◯	◯
Glucose simulator	◯	◯	◯	◯	◯	◯
Personal heart rate target calculation	◯	◯	◯	◯	◯	◯
Exercise plan	◯	◯	◯	◯	◯	◯

The information from ____ was relevant to me

	Not applicable	Strongly disagree	Disagree	Neither agree nor disagree	Agree	Strongly agree
Handout	◯	◯	◯	◯	◯	◯
Glucose simulator	◯	◯	◯	◯	◯	◯
Personal heart rate target calculation	◯	◯	◯	◯	◯	◯
Exercise plan	◯	◯	◯	◯	◯	◯

Please provide any feedback and comments you might have for improving this physical activity education session. ______________________________

Please rate each of the following statement regarding the device you used in this study.

Usability: This tool is easy to use and user friendly.

	Strongly disagree	Disagree	Neither agree nor disagree	Agree	Strongly agree
CGM	○	○	○	○	○
Fitbit	○	○	○	○	○

Convenience: This tool is convenient for me to use in my everyday life.

	Strongly disagree	Disagree	Neither agree nor disagree	Agree	Strongly agree
CGM	○	○	○	○	○
Fitbit	○	○	○	○	○

Value: This tool is useful and beneficial.

	Strongly disagree	Disagree	Neither agree nor disagree	Agree	Strongly agree
CGM	○	○	○	○	○
Fitbit	○	○	○	○	○

Relevance: This tool provides information that is of interest to me.

	Strongly disagree	Disagree	Neither agree nor disagree	Agree	Strongly agree
CGM	○	○	○	○	○
Fitbit	○	○	○	○	○

Motivating: I am motivated to use this tool to track my daily exercise-related behaviors.

	Strongly disagree	Disagree	Neither agree nor disagree	Agree	Strongly agree
CGM	○	○	○	○	○
Fitbit	○	○	○	○	○

Tech Support: There is adequate availability and quality of professional assistance throughout the use of this tool.

	N/A	Strongly disagree	Disagree	Neither agree nor disagree	Agree	Strongly agree
CGM	○	○	○	○	○	○
Fitbit	○	○	○	○	○	○

Confidence: I feel confident that I use this tool correctly.

	Strongly disagree	Disagree	Neither agree nor disagree	Agree	Strongly agree
CGM	○	○	○	○	○
Fitbit	○	○	○	○	○

Privacy: I am concerned about my privacy when using this tool.

	N/A	Strongly disagree	Disagree	Neither agree nor disagree	Agree	Strongly agree
CGM	○	○	○	○	○	○
Fitbit	○	○	○	○	○	○

Recommend: I would recommend this tool to my friends and family.

	Strongly disagree	Disagree	Neither agree nor disagree	Agree	Strongly agree
CGM	○	○	○	○	○
Fitbit	○	○	○	○	○

I like using this tool.

	Strongly disagree	Disagree	Neither agree nor disagree	Agree	Strongly agree
CGM	○	○	○	○	○
Fitbit	○	○	○	○	○

Please describe if you have any observations regarding your eating habits while using CGM. ____

What would you like to know more about your CGM data?

Please provide any feedback and comments you might have for future lifestyle interventions that use these tools. ____

During the past 12 months prior to your participation in this study, how often did you use health-related apps (e.g., Apple's Health, Google Fit, MyFitnessPal) on your smartphone?

- ◯ Everyday
- ◯ Couple times a week
- ◯ Less than once a month
- ◯ Never

Please list the health-related apps that you are currently using on your smartphone:

____

During the past 12 months prior to your participation in this study, have you ever used your smartphone to look up information about a health condition?

- ◯ Yes
- ◯ No

Have you ever owned a wearable health tracker (e.g., Fitbit, Apple Watch, Microsoft Band)?

- ◯ Yes
- ◯ No

Please list all the wearable health tracker(s) you have owned:

____

How likely or willing are you to use a wearable glucose sensor, like the one you used in this study, to help
you achieve your health and wellness goals (physical activity or weight management)?

- ◯ Very Likely
- ◯ Somewhat Likely
- ◯ Not Sure
- ◯ Somewhat Unlikely
- ◯ Very unlinkely

How likely or willing would you be to use a wearable glucose sensor to help you achieve your health and wellness goals (healthy eating or weight management), if the sensor did not have to be inserted under your skin (i.e., non-invasive)?

- ◯ Very Likely
- ◯ Somewhat Likely
- ◯ Not Sure
- ◯ Somewhat Unlikely
- ◯ Very Unlikely
