# Supplementary material for: Using Continuous Glucose Monitoring as a Biological Feedback Strategy to Motivate Physical Activity in Cancer Survivors: A Mixed-Methods Pilot Study
Source: Cancer Control. 2025 Jul 28;32:10732748251359406. doi: 10.1177/10732748251359406 (PMC12304624; doi:10.1177/10732748251359406)
Supplement: Supplemental Material - Using Continuous Glucose Monitoring as a Biological Feedback Strategy to Motivate Physical Activity in Cancer Survivors: A Mixed-Methods Pilot Study [file sj-pdf-2-ccx-10.1177_10732748251359406.pdf]

Survey Questions T1

Thank you for participating in our study!

This survey below asks for your perceptions and feelings about exercise. Please read each question carefully.

Please note that there are no right or wrong answers and no trick questions. You might feel some of the questions appear to be redudant. This is normal. We simply want to know how you personally feel about exercise. Your responses will be held in confidence and only used for our research purposes.

What is your sex at birth?

- ◯ Female
- ◯ Male

Please enter your date of birth (M-D-Y). ______________________________

What is your current marital status?

- ◯ Single, never married
- ◯ Married/co-habitating
- ◯ Separated
- ◯ Divorced
- ◯ Widowed

Are you of Hispanic or Latino origin?

- ◯ Yes
- ◯ No

Which category best describes your race or ethnicity?

- ◯ American Indian/Alaska Native
- ◯ Native Hawaiian/Pacific Islander
- ◯ Asian
- ◯ Black or African American
- ◯ White
- ◯ Other
- ◯ More than one race
- ◯ I prefer not to say

Please specify

____

Please specify

____

What is your highest level of education completed?

- ◯ Elementary school
- ◯ Middle school
- ◯ High school or GED
- ◯ Some college
- ◯ Vocational or associate degree
- ◯ Bachelor's degree
- ◯ Master's degree
- ◯ Doctoral degree
- ◯ I prefer not to say

What is your current employment status?

◯ Full-time
◯ Part-time
◯ Self-employed
◯ Unemployed
◯ Student
◯ Retired
◯ Other
◯ I prefer not to say

What was your household income before taxes last year?

◯ Less than $20,000
◯ $20,000 - $35,000
◯ $35,001 - $50,000
◯ $50,001 - $75,000
◯ $75,001 - $100,000
◯ Greater than $100,000
◯ I prefer not to say

Please specify

____

Regular Exercise is any planned physical activity (e.g., brisk walking, aerobics, jogging, bicycling, swimming, rowing, etc.) performed to increase physical fitness. Such activity should be performed 3 to 5 times per week for 20-60 minutes per session. Exercise does not have to be painful to be effective but should be done at a level that increases your breathing rate and causes you to break a sweat.

Considering the definition of exercise from the above paragraph, please indicate how strongly you agree or disagree with each of the following statements.

	Strongly Disagree	Disagree	Undecided	Agree	Strongly Agree
As far as I'm concerned, I don't need to exercise regularly.	○	○	○	○	○
I have been exercising regularly for a long time and I plan to continue.	○	○	○	○	○
I don't exercise and right now I don't care.	○	○	○	○	○
I am finally exercising regularly.	○	○	○	○	○
I have been successful at exercising regularly and I plan to continue.	○	○	○	○	○
I am satisfied with being a sedentary person.	○	○	○	○	○
I have been thinking that I might want to start exercising regularly.	○	○	○	○	○
I have started exercising regularly within the last 6 months.	○	○	○	○	○
I could exercise regularly, but I don't plan to.	○	○	○	○	○
Recently, I have started to exercise regularly.	○	○	○	○	○
I don't have the time or energy to exercise regularly right now.	○	○	○	○	○
I have started to exercise regularly, and I plan to continue.	○	○	○	○	○
I have been thinking about whether I will be able to exercise regularly.	○	○	○	○	○
I have set up a day and a time to start exercising regularly within the next few weeks.	○	○	○	○	○

I have managed to keep exercising regularly through the last 6 months.	○	○	○	○	○
I have been thinking that I may want to begin exercising regularly.	○	○	○	○	○
I have lined up with a friend to start exercising regularly within the next few weeks.	○	○	○	○	○
I have completed 6 months of regular exercise.	○	○	○	○	○
I know that regular exercise is worthwhile, but I don't have time for it in the near future.	○	○	○	○	○
I have been calling friends to find someone to start exercising with in the next few weeks.	○	○	○	○	○
I think regular exercise is good, but I can't figure it into my schedule right now.	○	○	○	○	○
I really think I should work on getting started with a regular exercise program in the next 6 months.	○	○	○	○	○
am preparing to start a regular exercise group in the next few weeks.	○	○	○	○	○
I am aware of the importance of regular exercise but I can't do it right now.	○	○	○	○	○

We are interested in the reasons underlying peoples' decisions to engage, or not engage in physical exercise. Using the scale below, please indicate to what extent each of the following items is true for you.

	(Not true for me) 0	1	(Sometimes true for me) 2	3	(Very true for me) 4
I exercise because other people say I should	○	○	○	○	○
I feel guilty when I don't exercise	○	○	○	○	○
I value the benefits of exercise	○	○	○	○	○
I exercise because it's fun	○	○	○	○	○
I don't see why I should have to exercise	○	○	○	○	○
I take part in exercise because my friends/family/partner say I should	○	○	○	○	○
I feel ashamed when I miss an exercise session	○	○	○	○	○
It's important to me to exercise regularly	○	○	○	○	○
I can't see why I should bother exercising	○	○	○	○	○
I enjoy my exercise sessions	○	○	○	○	○
I exercise because others will not be pleased with me if I don't	○	○	○	○	○
I don't see the point in exercising	○	○	○	○	○
I feel like a failure when I haven't exercised in a while	○	○	○	○	○
I think it is important to make the effort to exercise regularly	○	○	○	○	○
I find exercise a pleasurable activity	○	○	○	○	○
I feel under pressure from my friends/family to exercise	○	○	○	○	○
I get restless if I don't exercise regularly	○	○	○	○	○
I get pleasure and satisfaction from participating in exercise	○	○	○	○	○
I think exercising is a waste of time	○	○	○	○	○

Please indicate how sure you are that you will do each of the following:

	Not at all sure	A little sure	Pretty sure	Very sure
Exercise regularly (3 times a week for 20 minutes)	○	○	○	○
Exercise when you are feeling tired	○	○	○	○
Exercise when you are feeling under pressure to get things done	○	○	○	○
Exercise when you are feeling down or depressed	○	○	○	○
Exercise when you have too much work to do at home	○	○	○	○
Exercise when there are other more interesting things to do	○	○	○	○
Exercise when your family or friends do not provide any kind of support	○	○	○	○
Exercise when you don't really feel like it	○	○	○	○
Exercise when you are away from home (e.g., traveling, visiting, on vacation)	○	○	○	○

Please indicate how important each of the following statement is with respect to your decision to exercise or not to exercise.

	Not at all important	Slightly important	Moderately important	Very important	Extremely important
I think I would be too tired to do my daily work after exercising.	○	○	○	○	○
I would find it difficult to find an exercise activity that I enjoy that is not affected by bad weather .	○	○	○	○	○
I feel uncomfortable when I exercise because I get out of breath and my heart beats very fast.	○	○	○	○	○
Regular exercise would take too much of my time.	○	○	○	○	○
I would have less time for my family and friends if I exercised regularly.	○	○	○	○	○
At the end of the day, I am too exhausted to exercise.	○	○	○	○	○

Thinking about increasing your exercise level, please select a number on each row to answer these questions.

	(Never) 0	1	2	3	4	5	6	7	8	9	(Constantly) 10
Over the last week, how often did you feel you wanted to do it?	○	○	○	○	○	○	○	○	○	○	○
Over the last week, how often did you feel you needed to do it?	○	○	○	○	○	○	○	○	○	○	○
Over the last week, how often did you have a strong urge to do it?	○	○	○	○	○	○	○	○	○	○	○
Over the last week, how often did you imagine how good it would be to do it?	○	○	○	○	○	○	○	○	○	○	○
Over the last week, how often did you imagine how much better you'd feel if you do it?	○	○	○	○	○	○	○	○	○	○	○
Over the last week, how often did you imagine how much worse you'd feel if you didn't do it?	○	○	○	○	○	○	○	○	○	○	○
Over the last week, how often did you imagine yourself doing it?	○	○	○	○	○	○	○	○	○	○	○
Over the last week, how often did you imagine how you would do it?	○	○	○	○	○	○	○	○	○	○	○
Over the last week, how often did you imagine succeeding at it?	○	○	○	○	○	○	○	○	○	○	○
Over the last week, how often did you picture times you did something like this in the past?	○	○	○	○	○	○	○	○	○	○	○
Over the last week, how often did thoughts about it come to mind?	○	○	○	○	○	○	○	○	○	○	○
Over the last week, how often did other things remind you about it?	○	○	○	○	○	○	○	○	○	○	○
Over the last week, how often did thoughts about it grab your attention?	○	○	○	○	○	○	○	○	○	○	○

During a typical 7-Day period (a week), how many times on the average do you do the following kinds of exercise for more than 15 minutes during your free time.

STRENUOUS EXERCISE
(HEART BEATS RAPIDLY)
(e.g., running, jogging, hockey, football, soccer, squash, basketball, cross country skiing, judo, roller skating, vigorous swimming, vigorous long distance bicycling) ______________________

MODERATE EXERCISE
(NOT EXHAUSTING)
(e.g., fast walking, baseball, tennis, easy bicycling, volleyball, badminton, easy swimming, alpine skiing, popular and folk dancing) ______________________

MILD EXERCISE
(MINIMAL EFFORT)
(e.g., yoga, archery, fishing from river bank, bowling, horseshoes, golf, snow-mobiling, easy walking) ______________________

During a typical 7-Day period (a week), in your leisure time, how often do you engage in any regular activity long enough to work up a sweat (heart beats rapidly)?

- ◯ OFTEN
- ◯ SOMETIMES
- ◯ NEVER/RARELY

I plan to exercise regularly over the next week

- ◯ Strongly disagree
- ◯ Disagree
- ◯ Slightly disagree
- ◯ Neither disagree nor agree
- ◯ Slightly agree
- ◯ Agree
- ◯ Strongly agree

I intend to exercise regularly over the next week

- ◯ Strongly disagree
- ◯ Disagree
- ◯ Slightly disagree
- ◯ Neither disagree nor agree
- ◯ Slightly agree
- ◯ Agree
- ◯ Strongly agree

If I would exercise on a regular basis, then I would be doing something good for my health in future years

- ◯ Strongly disagree
- ◯ Disagree
- ◯ Slightly disagree
- ◯ Slightly agree
- ◯ Agree
- ◯ Strongly agree

If I would exercise on a regular basis, then there is also a short-term benefit for my health

- ◯ Strongly disagree
- ◯ Disagree
- ◯ Slightly disagree
- ◯ Slightly agree
- ◯ Agree
- ◯ Strongly agree

If I participate in regular exercise, then I will ...

	Strongly disagree	Disagree	Neither disagree nor agree	Agree	Strongly agree
Feel less depressed and/or bored	○	○	○	○	○
Improve my self-esteem	○	○	○	○	○
Meet new people	○	○	○	○	○
Lose weight	○	○	○	○	○
Build up muscle strength	○	○	○	○	○
Feel less tension and stress	○	○	○	○	○
Improve health or reduce risk of disease	○	○	○	○	○
Do better on my job	○	○	○	○	○
Feel more attractive	○	○	○	○	○
Improve heart and lung fitness	○	○	○	○	○
Gain muscle	○	○	○	○	○
Improve muscle tone	○	○	○	○	○
Feel better about my body	○	○	○	○	○
Increase energy level	○	○	○	○	○

The following questions relate to your usual sleep habits during the past month only. Your answers should indicate the most accurate reply for the majority of days and nights in the past month. Please answer all questions.

When have you usually gone to bed? ______________________________

How long (in minutes) has it taken you to fall asleep each night? ______________________________

What time have you usually gotten up in the morning? ______________________________

How many hours of actual sleep did you get at night? ______________________________

How many hours were you in bed? ______________________________

During the past month, how often have you had trouble sleeping because you

	Not during the past month	Less than once a week	Once or twice a week	Three or more times a week
Cannot get to sleep within 30 minutes	◯	◯	◯	◯
Wake up in the middle of the night or early morning	◯	◯	◯	◯
Have to get up to use the bathroom	◯	◯	◯	◯
Cannot breathe comfortably	◯	◯	◯	◯
Cough or snore loudly	◯	◯	◯	◯
Feel too cold	◯	◯	◯	◯
Feel too hot	◯	◯	◯	◯
Have bad dreams	◯	◯	◯	◯
Have pain	◯	◯	◯	◯

Other reason(s), please describe, including how often you have had trouble sleeping because of this reason(s): ____________________

	Not during the past month	Less than once a week	Once or twice a week	Three or more times a week
During the past month, how often have you taken medicine (prescribed or "over the counter") to help you sleep?	◯	◯	◯	◯
During the past month, how often have you had trouble staying awake while driving, eating meals, or engaging in social activity?	◯	◯	◯	◯
During the past month, how much of a problem has it been for you to keep up enthusiasm to get things done?	◯	◯	◯	◯

During the past month, how would you rate your sleep quality overall?

◯ Very good
◯ Fairly good
◯ Fairly bad
◯ Very bad
