# Supplementary material for: Using Continuous Glucose Monitoring as a Biological Feedback Strategy to Motivate Physical Activity in Cancer Survivors: A Mixed-Methods Pilot Study
Source: Cancer Control. 2025 Jul 28;32:10732748251359406. doi: 10.1177/10732748251359406 (PMC12304624; doi:10.1177/10732748251359406)
Supplement: Supplemental Material - Using Continuous Glucose Monitoring as a Biological Feedback Strategy to Motivate Physical Activity in Cancer Survivors: A Mixed-Methods Pilot Study [file sj-pdf-3-ccx-10.1177_10732748251359406.pdf]

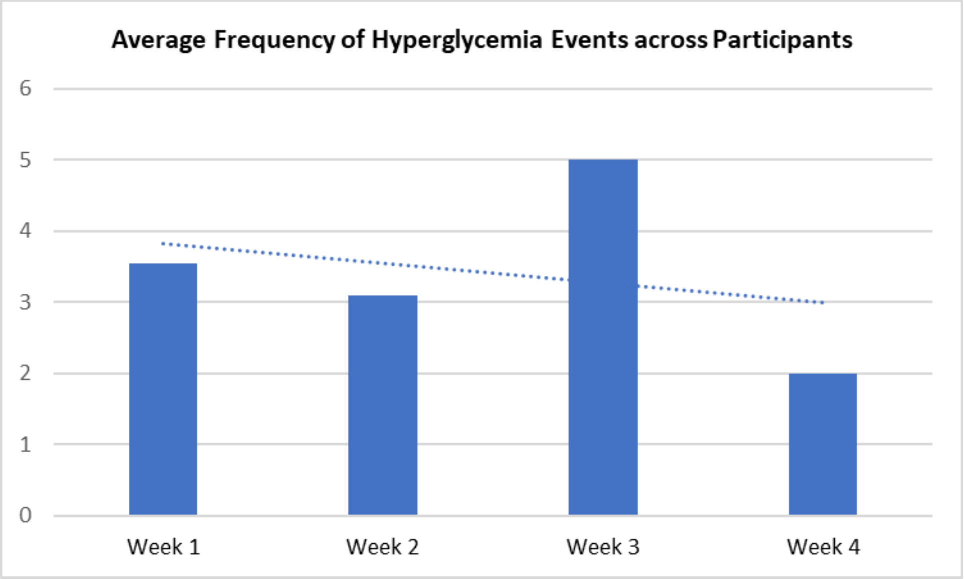

Average Frequency of Hyperglycemia Events across Participants
6
5
4
3
2
1
0
Week 1
Week 2
Week 3
Week 4

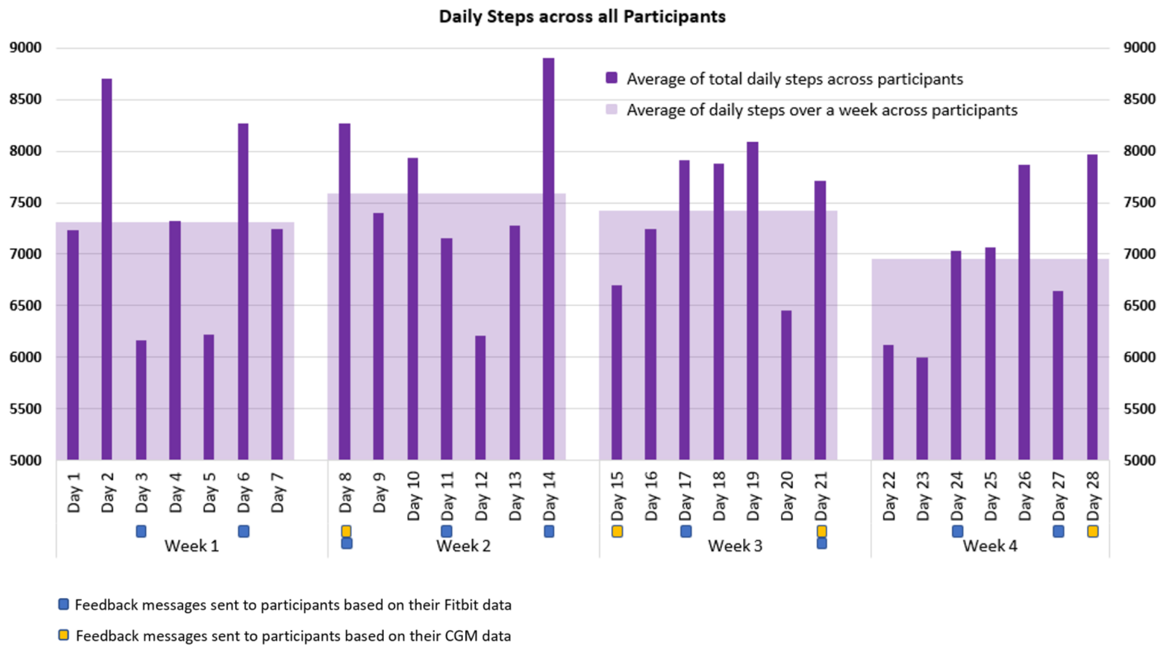

Daily Steps across all Participants
Average of total daily steps across participants
Average of daily steps over a week across participants
9000
8500
8000
7500
7000
6500
6000
5500
5000
Day 1
Day 2
Day 3
Day 4
Day 5
Day 6
Day 7
Day 8
Day 9
Day 10
Day 11
Day 12
Day 13
Day 14
Day 15
Day 16
Day 17
Day 18
Day 19
Day 20
Day 21
Day 22
Day 23
Day 24
Day 25
Day 26
Day 27
Day 28
Week 1
Week 2
Week 3
Week 4
Feedback messages sent to participants based on their Fitbit data
Feedback messages sent to participants based on their CGM data

Supplemental Table. Sample personalized biofeedback messages

Topic Category	Sample Text Message
**Goal setting**	▪ Hello ________, this is your first weekly CGM text message for the MyMoves study! Your average glucose level for the past week was ___ mg/dL. High glucose levels could increase your risk of developing type 2 diabetes. Keep in mind that whenever you move, your muscles burn blood glucose for fuel. So the more you move, the more glucose you burn! Let's see if your weekly average glucose level will be less than 100 mg/dL next week!
**Self-monitoring**	▪ Good morning/afternoon/evening, ____! Thank you for regularly scanning your glucose sensor. Let's take a quick look at your glucose patterns. In the past week, your average glucose level went below 100 mg/dL and was ___ mg/dL. Great job! Remember that aerobic exercise could have an immediate impact on lowering your glucose level. Next time when you observe a spike in your glucose, try a 10-minute brisk walk and see how it might change your glucose level.
**Outcome expectations & self-reflection**	▪ Hello ____, it's time to review your glucose data! In the past week, your average glucose level was above 100 mg/dL and was ___ mg/dL. Your previous week's glucose average was ____ mg/dL. It is normal that your glucose level fluctuates throughout the day, especially after meals. Can you think of anything that you might have done differently in the past week that might have led to this change in your weekly average glucose level?
